# Caffeine intake modulates the functioning of the attentional networks depending on consumption habits and acute exercise demands

**DOI:** 10.1038/s41598-019-46524-x

**Published:** 2019-07-11

**Authors:** Florentino Huertas, Esther Blasco, Consuelo Moratal, Juan Lupiañez

**Affiliations:** 1 Department of Physical Education & Sport Sciences, Catholic University of Valencia “San Vicente Mártir”, Valencia, Spain; 2 Department of Experimental Psychology, Mind, Brain, and Behavior Research Center (CIMCYC), University of Granada, Granada, Spain

**Keywords:** Attention, Cognitive control

## Abstract

Consume of stimulants (as caffeine) is very usual in different contexts where the performers have to take quick and accurate decisions during physical effort. Decision-making processes are mediated by the attentional networks. An experiment was carried out to examine the effect of caffeine intake on attention (alerting, orienting, and executive control) as a function of consumption habit under two physical exertion conditions (rest vs. aerobic exercise). Two groups of participants with different caffeine consumption profiles (moderate consumers vs. low consumers) performed the Attention Network Test–Interactions under four different conditions regarding activity (rest vs. exercise) and intake (caffeine vs. placebo). Results showed that whereas exercise led to faster reaction times (RT) in all cases, caffeine intake accelerated RT but only at rest and in moderate caffeine consumers. More importantly, caffeine intake reduced the alertness effect in moderate consumers only at the rest condition. No interactions between Intake and Activity were observed in the other attentional networks, with exercise reducing orienting independently of caffeine intake, which suggests that physical exercise and caffeine are different modulators of attention but can interact. Caffeine intake had differential effects on reaction speed at rest and during physical exercise depending on the individual consumption habit. On the basis of these finding it seems that mainly alertness is modulated differently by internal and external “arousing” conditions.

## Introduction

At different environmental situations (as practicing sports, driving vehicles, surgery interventions…) people must often perform physical activities and respond quickly to external challenges. Decision-making processes are mediated by the three attentional networks (alerting, orienting, and executive control) defined by Petersen & Posner^1^.

The executive control network is involved in situations that require planning, decision making, error detection, execution of novel responses, or overcoming habitual actions^2^. The orienting network is responsible for allocating attention to a particular object or region of space in a voluntary-endogenous or reflexive-exogenous way, enhancing its processing while ignoring irrelevant objects/locations^3^. Finally, the alerting network participates in the general activation of cortical and thalamic areas, thus preparing the perceptual-motor system for fast reactions through changes in the norepinephrine system.

During different situations of daily living like working and sport tasks, these attentional networks have to work in cooperation to maintain an efficient psychomotor performance during the physical-cognitive dual task challenges inherent to those activities.

The relationship between exercise and two of the main core components of executive control (inhibitory processes and cognitive flexibility) has been widely studied by researchers. Inhibitory control refers to the ability to attend to a relevant stimulus while ignoring the others that are not relevant to the goal or task at hand. Cognitive flexibility refers to the ability to rapidly switch between different tasks. Previous research reported that the above- mentioned component of executive control were improved (enhancing inhibitory processes^4^ and reducing switch cost^5^) when participants were exercising under an aerobic workload. Yet, and according to the hypofrontality hypothesis^6^, neuroelectric^7^ and behavioral^8^ findings showed impaired conflict resolution during exercise conditions as compared to rest conditions. In addition, other studies failed to show any differential effects of acute aerobic exercise on participants’ performance^9^, thus adding more contradictory findings regarding the nature and direction of this relationship. More importantly for the purposes of the present study, scarce studies have analyzed the effect of concomitant physical effort on the three attentional networks assessed at the same time. Faster RT during the exercise condition were reported by Chang and colleagues^10^ using the Attentional Network test (ANT)^11^, but only on trials with incongruent flankers, thus leading to reduced interference^10^. Another study, closer to the present one, using a version of the ANT named Attentional Network Test Interaction (ANT-I)^12^ showed no effect of exercise on executive control^13^.

Regarding spatial attentional orienting, in a series of studies Pesce and colleagues found that aerobic exercise enhanced the flexibility in modulating the spatial extent of visual attentional focus at different ages and sport modalities^14–18^. Regarding the shifting of spatial attention in the visual space, the ability we will analyze in our study, previous findings have shown that the effect of exercise on cueing effects in visuospatial attention is modulated by gender^19^ and sport expertise^20^. However, to the best of our knowledge, few studies have explored the effects of acute bouts of aerobic exercise on the deployment of exogenous visual spatial attention. Previous studies found that an acute bout of aerobic exercise performed during or even immediately before a spatial orienting task eliminated the typical spatial cueing effect^21^. Later studies established that spatial orienting in overt attentional capture was influenced by high intensity exercise, and depended on participants’ fitness level showing more reduced attentional effects in low-fit participants than in high-fit participants^22^. However, contradictory results were found when attentional networks were assessed simultaneously, with no effects of exercise on exogenous spatial orienting^10,13^.

On the other hand, the modulation of alertness by acute exercise has been widely studied but also shows mixed results. There are two types of alertness. Phasic extrinsic alertness is associated with an abrupt increase of nonspecific activation when a warning cue is presented preceding the target (this is related to the orienting response^23^). By contrast, tonic alertness or vigilance refers to sustained activation over a period of time^24^. Regarding phasic alertness, previous studies reported that participants’ accuracy was lower when the task was performed under an intense physical exertion condition than at rest^25^. However, contradictory results emerge again when attentional networks are assessed simultaneously using the ANT task. Neuroelectric findings have showed larger P3 amplitude (a positive-going component associated to the amount of attentional resources allocated to environmental events), on alerting trials in the exercise condition^10^. However, another behavioral study has found a reduction of the size of the phasic alerting effect during aerobic exercise as compared to the rest condition^13^. Regarding the tonic alertness (sustained attention), there is a broad consensus that moderate to intense exercising enhance the state of tonic vigilance^26^, though poorer vigilance performance was observed under heavier exercising conditions^27^.

Previous research has shown that the effect of acute bout of exercise on attentional networks seems to be moderated by different individual and contextual constraints (e.g., participant physical fitness, sport modality, exercise intensity, or others as consume of ergogenic or stimulant substances). Therefore these variables need to be considered in the study of the acute exercise-attention relationship.

During challenging environmental conditions, people often decide to consume stimulants to improve performance. Caffeine (1,3,7-trimethylxanthine) has become one of the most popularly used ergogenic substances to improve both physical and cognitive performance^28,29^. However, though improvement in general attentional performance induced by caffeine intake is well accepted, there is scarce and controversial evidence about its effect on each attentional network and their interactions.

Regarding the effect of caffeine intake on executive control, caffeine has been shown to have a positive influence on the ability to switch attention between tasks (task switching) and anticipatory control processes^30,31^. However caffeine consumption has failed to modulate other facets of inhibitory function like the ability to control interference from distracting stimuli (using Ericksen Flanker Test) and the ability to suppress responses selectively (e.g., by using a Stroop task)^32^. Previous studies exploring the interactions between exercise and caffeine on executive control reported a positive effect of caffeine intake after exercise^33^, during aerobic^34^ and intermittent exercises^35^.

Few studies have been nevertheless conducted on the effect of caffeine on attentional orienting^36,37^. Such studies, performed under resting conditions, have only shown marginal effects of caffeine intake on orienting when low caffeine consumers were tested. However, controversial findings have been reported when interaction between caffeine intake and orienting was studied manipulating exercise conditions. While some studies did not find modulations of caffeine on object tracking or covert spatial orienting respectively after aerobic acute exercise^38,39^, others showed positive effects on shifting attention after longer medium to high intensity aerobic exercises^33^.

Considering alertness, systematic reviews have reported significant enhancements of various doses of caffeine on vigilance^40,41^. Concerning the interaction between caffeine and exercise on alertness, sustained vigilance appears to be improved by caffeine intake after^33^ and during^34^ an acute exercise bout. However, caffeine specific effects on phasic alertness during exercise hasn’t been studied yet.

Scarce studies have investigated the effects of caffeine on the functioning of the three attentional networks concurrently. To our knowledge, Brunyé and colleagues are the only ones who have investigated the effects of caffeine intake on the three attentional networks simultaneously by means of the ANT task^36,37^. They have reported that caffeine-induced physiological arousal amplifies global spatial processing, vigilance and executive control, but these effects are at least partially driven by the administered dose and habitual caffeine consumption. These findings are in line with those showing that caffeine increases arousal on every type (low and habitual) of caffeine consumers^42^. However, most studies in this topic have involved caffeine–deprived habitual caffeine consumers, which makes it difficult to know whether the observed findings are due to the effects of caffeine intake or the alleviation of caffeine withdrawal. On this vein, another study investigated acute effects of caffeine in both habitual and non-habitual caffeine consumers observing a more positive effect of caffeine intake on consumers’ mood state while improving cognitive performance more in the non-consumers^43^. Thus, previous findings suggest that the effects of caffeine intake on attentional performance could be explained, not only by a withdrawal reduction model, but also by other mechanisms involving the brain biochemistry and the effect of caffeine on dopamine-rich areas of the brain.

Considering that in many daily life activities caffeine intake is combined with a physical effort, we have to take into account the evidence from previous literature reviewing the effects of acute exercise on cognition from a neurochemical perspective. A recent review by McMorris^44^ has shown that long duration, moderate intensity, and heavy exercise generate excessive concentrations of catecholamines and cortisol inhibiting working memory. Nevertheless, heavy exercise has a positive effect on long-term memory due to activation of β-adrenoreceptors and increased exercise-induced brain-derived neurotrophic factor (BDNF) levels. Since it is widely known that attentional functioning is also modulated by the exercise- induced physiological response facilitating or inhibiting attentional performance, it would be worth to study the joint effects of physical exercise and caffeine as both take part in similar mechanism underlying the brain biochemistry.

However, and importantly for our research, the exercise- and caffeine-induced changes on the attentional networks are somewhat equivocal^44^, as described above, and therefore future research is necessary. Furthermore, understanding the interplay between exercise, caffeine intake and consumption habits on the functioning of the three attentional networks is crucial for adapting caffeine consumption to optimize its expected benefits. Nevertheless, to the best of our knowledge, no study so far has examined this interaction. In order to fill this gap, the goal of the present study was to explore the individual and interactive effects of caffeine intake and acute physical exercise on the functioning of the attentional networks in the context of a multi-functional task (ANT-I task^12^) under four different conditions resulting from the combination of activity (i.e., rest vs. sustained moderate aerobic exercise) and intake (caffeine vs. placebo).

Following the above reviewed literature, behavioral and neurophysiological findings lead to argue that the efficiency of the central nervous and cognitive function (reaction time and attention) is modulated by exercise-induced^44^ and caffeine-induced^37^ neurobiochemical mechanisms regulating arousal. It is relevant to highlight that caffeine consumption^41^ and exercise^45^ modulate the baseline arousal level (lower under rest and placebo conditions), mainly in participants more habituated to caffeine intake. Based on this evidence we expected to observe an interaction between caffeine intake, consumption habit and exercise condition on cognitive performance. This interaction was expected for overall RT and alertness, as these variables seems to be affected by opposite manipulations reducing arousal^46–48^. In our case, we expected faster RT (tonic alertness) and reduced phasic alertness effects in the conditions in which the combination of exercise and caffeine intake would lead to stronger activation. We also wanted to explore whether caffeine intake and exercise also affect the functioning of the other attentional networks (orienting and executive control). Although some effects have been previously reported in the above reviewed literature, no clear predictions were anticipated.

## Method

### Participants

Twenty-four male undergraduate sport sciences students (age range: 21–25 years; *M* = 22 years) were selected to participate in this study. We used the usual sample size with the ANTI task^12,49^, without using a priori power analyses. Therefore, a sensitivity analysis was conducted using G*power^50^ which showed that with our sample size (N = 24) and 48 repeated measures, the minimum effect size that could have been detected for α = 0.5, and 1 − β = 0.80, for 2 groups, is f = 0.11 (minimum detectable effect).

Participants were assigned to one of two groups according to their habitual caffeine consumption based on their responses to a self-report food survey. Following previous recommendations^51^ we used a between-group design, allocating participants to one of two groups depending on their caffeine consumption habit and dispensing an individual caffeine dosage depending on participants’ body weight. According to participants’ estimated daily caffeine intake^52^, they were assigned to either the low or moderate caffeine consumers group using a cut-off point criterion (Low consumers ≤ 50 mg/day vs. Moderate consumers > 50 mg/day)^43^. Half of participants were categorized as low caffeine consumers (*M* = 14 mg/day, range = 0–28 mg/day) and half were considered moderate caffeine consumers (*M* = 101 mg/day, range = 57–228 mg/day). All participants were non-smokers who were in good health and were not taking any medication. They all completed both an ethical clearance form and a statement regarding informed consent.

### Procedure

Present research followed accepted ethical, scientific and medical standards and was conducted in compliance with recognized international standards, in accordance with the revised version (2013) of the Helsinki Declaration. Informed consent was obtained from all study participants. The study protocol was approved by the Ethics Committee of the Catholic University of Valencia, Spain (UCV/2016-2017/02).

**Figure 1:**
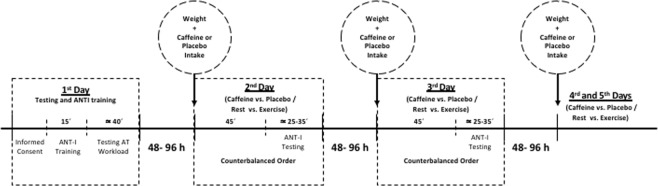
Schematic representation of the experimental protocol timing.

Participants visited the laboratory on five separate days with a minimum interval of 48 hours and a maximum interval of 96 hours, approximately at the same time of the day. They were instructed to abstain from consuming caffeine or any stimulant substance and to avoid physical exercise for 24 h prior to each session. On the first visit, participants performed a graded submaximal test in order to determine their Lactate Threshold (LT). The next four visits were experimental sessions in which participants had to complete the attentional tasks corresponding to the Intake (caffeine vs. placebo) and Activity (rest vs. exercise) conditions, whose order was counterbalanced across participants using a Latin square design. The experimental timeline is depicted in Fig. 1. At the end of the last experimental session, participants were debriefed on the purposes of the study and received a description of their physical and cardiovascular fitness and attentional performance.

During cycling parameter estimation test and attentional evaluation we have employed the same attentional tasks, and similar resources and procedures previously described by the authors in a similar research about the effect of acute bouts of exercise on functioning of the attentional networks^13^.

#### Cycling parameter estimation test

On the first session, participants were informed about the details of the study and gave written informed consent. After that, participants practiced a reduced version of the ANT-I. Next, participants were fitted with a Polar RS800 heart rate monitor (Polar Electro Ltd., Kempele, Finland). A magnetically braked cycle ergometer (Cardgirus Medical, G&G Innovación, La Bastida, Alava, Spain) was adjusted to accommodate them to perform a graded submaximal test according to previous recommendations^53^. After a 7-min warm-up, the test started at an initial workload of 100 W, with increments of 30 W every 4 min. The test was terminated when participants acknowledged voluntary exhaustion, could not maintain the minimum cadence of 60 revolutions per minute (rev · min^−1^) or when their heart rate (HR) reached 95% of their maximum HR (HR_max_) obtained from the age-predicted equation^54^: HR_max_ = 208 − 0.7 · Age. Finally, participants pedaled until their HR was under 120 beats per minute (bpm) before getting off the cycle ergometer. Earlobe capillary blood samples were collected in the last 15 s of each stage and were analyzed by a blood lactate test meter (Lactate Pro LT-1710, Arkay KDK, Japan). Power output and HR were continuously monitored using a sampling rate of 1 Hz. Determination of the LT was based on the criteria established by previous studies^55^. Workload at LT was defined as the power output elicited at the stage before LT. HR at LT was estimated as the statistical mode value of HR at the stage before LT. These results were used to set the individual exercise workloads in the exercising experimental conditions.

#### Materials and procedure used in the experimental sessions

Prior to each experimental session and upon their arrival in the lab, participants were weighed and given a capsule containing their previously assigned intake substance. We used a double-blind design to manipulate this variable. Caffeine (4 mg · kg ^−1^ pure anhydrous powder) and placebo (pregelatinized starch powder) were administered in identical color, size, weight, and shape capsules with *ad libitum* water. Dosage was selected based on a previous review^51^ which suggested doses from 3 to 6 mg · kg^−1^, and considering the recommendations by European Food Safety Authority^56^. Next, participants were asked to take a 45-minute break to allow for sufficient plasma concentrations of caffeine after consumption^57^. Participants completed the ANT-I task at approximately 60 cm from the computer monitor in a dimly-lit laboratory. A headphone set was used to deliver the acoustic alerting signal. Two computers were used simultaneously in the experimental set-up. One was used to run E-Prime software^58^ presenting stimuli and collecting the participants’ responses during the attentional task in all experimental conditions. The other computer was used to run Cardgirus® software adjusting and collecting power output and HR data during exercise conditions. In the rest condition, participants completed ANT-I while sitting on a chair.

Previous findings have shown that the efficiency of some brain areas and systems involved in cognitive arousal and cognitive resources is related with physiological exercise workload-induced changes^44,45^. During the exercise conditions, participants performed the attentional task while cycling on the ergometer at 80% of their workload at LT. This intensity has been used in previous studies investigating the effect of acute exercise on attentional networks^13^, in which physiological variables (e.g., HR blood flow and lactate) were maintained in a moderate aerobic steady-state zone during the whole session. With the aim of keeping physiological response (HR) in a steady-state zone, a 10-min incremental warm-up was used to reach the initial predetermined exercise workload. Workload could be regulated lightly by the experimenter manually throughout the exercise sessions to maintain each individuals’ HR in the target steady-state zone. To confirm that participants performed the cognitive task under a steady state metabolic during both rest and exercise sessions, blood was drawn from their earlobes for lactate analysis at the beginning and immediately after finishing the attentional task. Moreover, HR was monitored during the whole sessions.

#### Attentional task (ANT-I)

**Figure 2:**
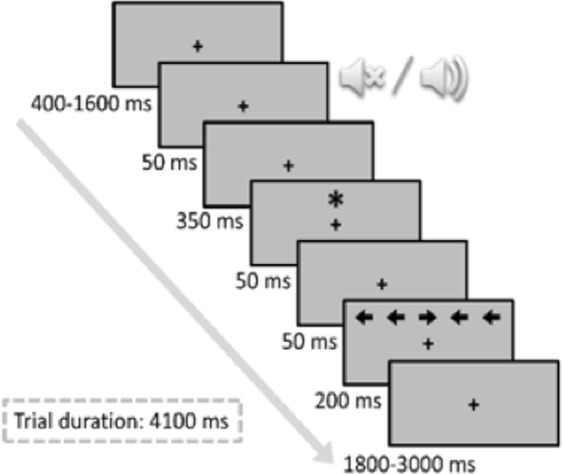
Procedure of the ANT-I task.

The participants’ task was to respond as quickly and accurately as possible by pressing the left or right key placed on the handlebar. Participants responded to the direction of the target stimulus (a central target arrow 0.55° long pointing either left or right), which was flanked by two identical to the target irrelevant arrows on each side (0.06° away from each other). On each trial, an acoustic alerting tone (2000 Hz and 50 ms) and/or spatial orienting visual cue (an asterisk 0.6° long and 50 ms) preceded the target arrow. Participants were strongly encouraged to keep their eyes fixed on the fixation point (variable duration of 400–1600 ms) throughout the trial. The sequence of events for each trial is shown in Fig. 2. The interference variable was defined according to the congruency of the direction of the flankers and target arrows: congruent trials (50% of trials), when the target was flanked by arrows pointing in the same direction, and incongruent trials (the other 50% of trials), when the flanking arrows and the target pointed in opposite directions. The orienting signal was presented in two thirds of the trials above or below the fixation point. Three orienting conditions were thus established according to the presence of the cue; cued location trials, when the cue was presented at the same location as the target; uncued location trials, when the cue was presented at the opposite location to the target, or absence of cue, no-cue trials, when the cue was not presented. The alerting signal was presented before the onset of the target in only half of the trials. The alerting variable was established according to the presence (tone) or absence (no tone) of the alerting sound. The target was presented until participants responded or for 1800 ms. After the response, or after the maximum time had elapsed, the fixation point was presented for a variable duration (depending on the RT of the preceding trial and the duration of the initial display for that trial) so that all trials were equally long. Initially, participants completed a practice block of 48 trials, followed by five experimental blocks of 48 trials each, with resting intervals of about 1 min between them, but maintaining constant cycling parameters in the exercise sessions.

Thus, participants performed the ANT-I for approximately 25 min in each experimental session. The exercise sessions required longer time (about 35 min) because the warm-up stage was necessary to reach the physiological steady state to perform the 25 min of continuous cycling at the pre-established HR and exercise workload. Both the workload intensities and the duration made it possible to obtain the required amount of data from the attentional task during exercise, maintaining a physiological steady state while avoiding fatigue in the participants. Results regarding physiological response to each Activity and Intake condition confirm the high level of stability of the variables used for measuring physiological response to exercise.

#### Design and statistical analysis

We conducted a mixed repeated-measures MANOVA on the data of each dependent variable (HR, RT, and accuracy), with caffeine Consumption Habit (low, moderate) as a between-group factor, and the other independent variables (Intake: caffeine, placebo; Activity: rest, exercise; Alerting: tone, no tone; Orienting: uncued, no cue, cued; and Congruency: congruent, incongruent) as within- participant factors. Subsequent ANOVAs were performed for mean RT and overall error percentages to analyze the general effects of Consumption Habit, Intake, and Activity on each attentional function. Post hoc analyses (paired *t*-tests) were conducted to further explore significant interactions.

## Results

### Preliminary analysis

Descriptive statistics of anthropometric and physiological characteristics of the whole sample and separately for low and moderate caffeine consumers are displayed in Table 1. No significant differences were observed between groups in none of these variables (all *p*s > 0.2).

**Table 1:** Anthropometric and physiological characteristics according to participant group.

Variables	Mean ± *SD*		All
	Low consumers	Moderate consumers	
Age (years)	21.9 ± 1.9	22.9 ± 3.9	22.4 ± 3.0
Weight (kg)	74.6 ± 6.5	72.9 ± 11.1	73.7 ± 9.0
Height (cm)	178.2 ± 4.6	178.8 ± 5.7	178.5 ± 5.1
HR baseline (bpm)	64 ± 6	67 ± 8	66 ± 7
Predicted HR_max_ (bpm)	177 ± 3	183 ± 2	180 ± 2
HR at LT (bpm)	150 ± 13	155 ± 9	155 ± 11
LT (mmol · L^−1^)	3.6 ± 0.8	3.6 ± 0.7	3,6 ± 0.8
Workload at LT (W)	173.6 ± 28.0	160.0 ± 22.1	166.5 ± 25.5

Note. *SD* = standard deviation; HR = heart rate; LT = lactate threshold; bpm = beats per minute; W = watts; mmol · L^−1^: millimoles per liter; HR_max_ = maximum predicted heart rate.

The Mean *(M)* and Standard Deviation *(SD)* of HR and lactate values per experimental session are displayed in Table 2.

**Table 2:** Mean (M) ± SD values of workload and physiological response values to each Activity and Intake experimental condition according to participants’ caffeine consumption habit.

Variables	Rest						Exercise					
	Caffeine			Placebo			Caffeine			Placebo		
	Low	Moderate	All	Low	Moderate	All	Low	Moderate	All	Low	Moderate	All
Workload (W)	—	—	—	—	—	—	106.3 ± 19.3	104.7 ± 19.6	105.5 ± 19.0	106.7 ± 23.6	101.5 ± 18.1	104.0 ± 20.6
Initial [La−] (mmol · L^−1^)	—	—	—	—	—	—	2.5 ± 0.9	2.4 ± 1.1	2.4 ± 1.0	2.2 ± 0.7	2.3 ± 0.8	2.3 ± 0.7
Final [La−] (mmol · L^−1^)	—	—	—	—	—	—	2.0 ± 0.9	2.4 ± 0.8	2.2 ± 0.8	1.9 ± 0.8	2.2 ± 0.7	2.1 ± 0.7
HR (bpm)	67 ± 8	70 ± 11	68 ± 9	64 ± 6	67 ± 8	65 ± 7	120 ± 9	126 ± 8	122 ± 8	121 ± 7	126 ± 9	123 ± 8
% predicted HR_max_	38.5 ± 5.2	38.2 ± 6.0	38.3 ± 5.6	36.2 ± 4.1	37.7 ± 5.4	36.5 ± 4.0	68.6 ± 4.0	69.2 ± 3.7	68.7 ± 4.5	68.6 ± 4.8	69.3 ± 3.3	68.8 ± 4.1
% HR at LT	44.1 ± 5.2	45.5 ± 4.0	44.7 ± 5.3	43.6 ± 5.4	43.8 ± 4.6	43.7 ± 5.3	80.2 ± 4.0	85.3 ± 4.3	82.3 ± 4.1	80.3 ± 4.1	85.2 ± 4.3	82.6 ± 4.1

Note. Note that under Rest condition the Workload and Lactate values were -, because no work was developed and the instrument used to measure Lactate concentration does not give values below 0.8. *SD* = standard deviation; HR = heart rate; HR_max_ = maximum predicted heart rate; LT = lactate threshold; bpm = beats per minute; W = watts; mmol · L^−1^: millimoles per liter.

In order to confirm whether the applied exercise workload and caffeine intake induced different changes in physiological state in each intake group of participants, a mixed 2 (Habit) × 2 (Intake) × 2 (Activity) MANOVA was performed on participants’ average HR values, with Habit as a between-group factor. Results showed a significant main effect of Activity, *F*(1, 22) = 4158.19, *p* < 0.001, η^2^ = 0.99, indicating that HR increased by 56 bpm from the rest to the exercise condition. However, caffeine intake did not modulate HR (*p* = 0.321). Similarly, Consumption Habit did not modulate HR response under either Activity (*p* = 0.238) or Intake (*p* = 0.841) conditions.

### Attentional networks functioning

Incorrect responses (1.63%), and those with RT faster than 200 ms (considered as anticipations) or slower than 3 SD above the overall mean (1.28%; considered as lapses) were discarded from the analysis of RT. Mean RTs were computed with the remaining RTs and submitted to the corresponding analyses. Mean RTs and error percentages per experimental condition are displayed in Table 3.

**Table 3:** Mean Reaction Time and error percentage (in parenthesis) for each experimental condition and for each consumption habit group.

			Low Consumers						Moderate Consumers					
			No Tone			Tone			No Tone			Tone		
			Uncued	No Cue	Cued	Uncued	No Cue	Cued	Uncued	No Cue	Cued	Uncued	No Cue	Cued
Exercise	Caffeine Intake	Congruent	451	467	428	445	427	422	455	470	440	441	429	414
			(0.00%)	(0.69%)	(0.17%)	(0.35%)	(0.69%)	(0.00%)	(0.00%)	(0.35%)	(0.00%)	(0.35%)	(0.35%)	(0.00%)
		Incongruent	520	490	480	533	497	474	532	530	505	540	508	482
			(6.42%)	(1.39%)	(1.74%)	(5.90%)	(2.78%)	(2.43%)	(5.56%)	(2.08%)	(1.04%)	(5.56%)	(2.43%)	(1.04%)
	Placebo Intake	Congruent	445	464	426	446	429	410	456	468	432	441	432	414
			(0.69%)	(0.69%)	(0.69%)	(0.52%)	(0.69%)	(0.17%)	(1.04%)	(0.69%)	(0.00%)	(0.35%)	(0.35%)	(0.00%)
		Incongruent	524	517	495	535	495	481	540	523	502	532	500	481
			(3.30%)	(3.30%)	(2.26%)	(6.77%)	(1.91%)	(4.34%)	(2.43%)	(4.51%)	(2.43%)	(7.29%)	(2.43%)	(3.13%)
Rest	Caffeine Intake	Congruent	505	509	460	496	472	456	496	500	468	484	462	447
			(0.17%)	(0.52%)	(0.00%)	(0.35%)	(0.00%)	(0.00%)	(0.00%)	(0.69%)	(0.00%)	(0.35%)	(0.00%)	(0.00%)
		Incongruent	581	559	520	585	542	510	586	565	530	591	546	510
			(3.30%)	(0.87%)	(0.52%)	(5.03%)	(2.43%)	(0.87%)	(2.43%)	(1.04%)	(0.35%)	(5.21%)	(2.43%)	(1.04%)
	Placebo Intake	Congruent	493	510	466	488	473	456	536	547	508	520	490	486
			(0.17%)	(0.17%)	(0.17%)	(0.00%)	(0.35%)	(0.35%)	(0.00%)	(0.00%)	(0.00%)	(0.00%)	(0.00%)	(0.35%)
		Incongruent	576	555	528	580	536	511	631	620	565	615	567	544
			(2.26%)	(2.26%)	(1.39%)	(4.51%)	(2.26%)	(2.43%)	(1.74%)	(2.78%)	(1.04%)	(2.78%)	(2.43%)	(2.43%)

A first analysis was carried out on the Mean RTs and Error percentages to test the general functioning of the ANTI task in this population. The 2 (Alerting) × 3 (Orienting) × 2 (Congruency) repeated measures ANOVA performed on Mean RTs showed the usual results, i.e., main effects for each variable, *F*(1, 23) = 55.41, *p* < 0.001, η^2^ = 0.71, *F*(2, 46) = 165.36, *p* < 0.001, η^2^ = 0.88, and *F*(1, 23) = 628.54, *p* < 0.001, η^2^ = 0.96 (for Alerting, Orienting and Congruency respectively), and the usual Alerting × Orienting, *F*(2, 46) = 52.97, *p* < 0.001, η^2^ = 0.70 (larger cueing effect with the alerting signal), Alerting × Congruency, *F*(1, 23) = 25.33, *p* < 0.001, η^2^ = 0.52 (larger flanker-congruency effect with the alerting signal) and Orienting × Congruency interactions, *F*(2, 46) = 35.86, *p* < 0.001, η^2^ = 0.61 (smaller flanker-congruency effect for cued than uncued trials).

The corresponding analysis performed on the error percentages showed similar results with *F*(1, 23) = 3.98, *p* < 0.058, η^2^ = 0.15, *F*(2, 46) = 11.96, *p* < 0.001, η^2^ = 0.34, and *F*(1, 23) = 32.57, *p* < 0.001, η^2^ = 0.59 for the main effects of Alerting, Orienting and Congruency respectively. Similarly, the usual Alerting × Congruency and Orienting × Congruency interactions were also significant, *F*(1, 23) = 7.10, *p* < 0.014, η^2^ = 0.24, and *F*(2, 46) = 13.06, *p* < 0.001, η^2^ = 0.36, respectively (the Alerting × Orienting did not reach statistical significance, *F*(2, 46) = 1.92, *p* < 0.158, η^2^ = 0.08).

Importantly, when Consumption habit, Intake, and Activity were added to the analysis, these variables modulated the main effect of some of the attentional variables, but no interaction between attentional variables was modulated. Therefore, in order to have a closer look to the data, 4 different ANOVAs were performed, one for Mean RT (and overall error percentages) to analyze the general effects of Consumption habit, Intake, and Activity, and one for each attentional network to analyze how they affect attentional performance.

#### Overall effects of Consumption habit, Intake, and Activity

**Figure 3:**
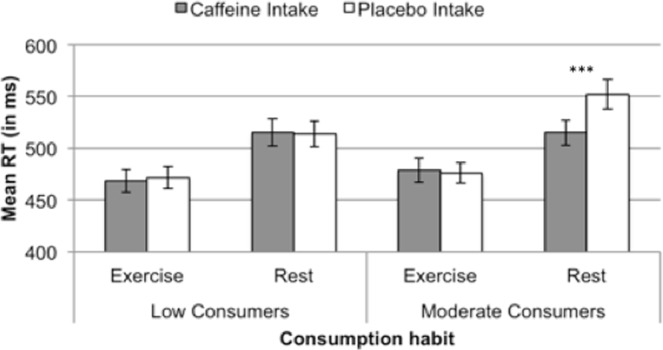
Mean RT as a function of Activity and Caffeine intake for each group of Consumption habit. Note: ***p < 0.005.

A 2 (Consumption habit) × 2 (Intake) × 2 (Activity) mixed ANOVA was performed on Mean RT and percentage of errors, with Consumption habit as a between-participants factor and the other variables as within participants variables. RT results revealed a significant main effect of Activity, *F*(1, 22) = 91.70, *p* < 0.001, η^2^ = 0.81, with 50 ms faster RT in the exercise than the rest condition. Although Intake did not modulate significantly RT, both the Activity × Intake and the three-way Activity × Intake × Consumption habit interactions were significant, *F*(1, 22) = 4.86, *p* = 0.038, η^2^ = 0.18, and *F*(1, 22) = 7.74, *p* = 0.011, η^2^ = 0.26, respectively. Partial ANOVAs showed that the effect of Intake was only observed at rest in moderate caffeine consumers, *F*(1, 11) = 16.68, *p* = 0.002, η^2^ = 0.60 (F < 1 in the remaining cases). As can be observed in Fig. 3, whereas exercise led to faster RT in all cases, caffeine intake led to 37 ms faster RT but only at rest and in moderate caffeine consumers.

The corresponding analysis on the error percentages only revealed a main effect of Activity, *F*(1, 22) = 19.65, *p* < 0.001, η^2^ = 0.47, showing that participants made slightly more errors (0.73%) in the exercise than the rest condition.

#### Alertness

**Figure 4:**
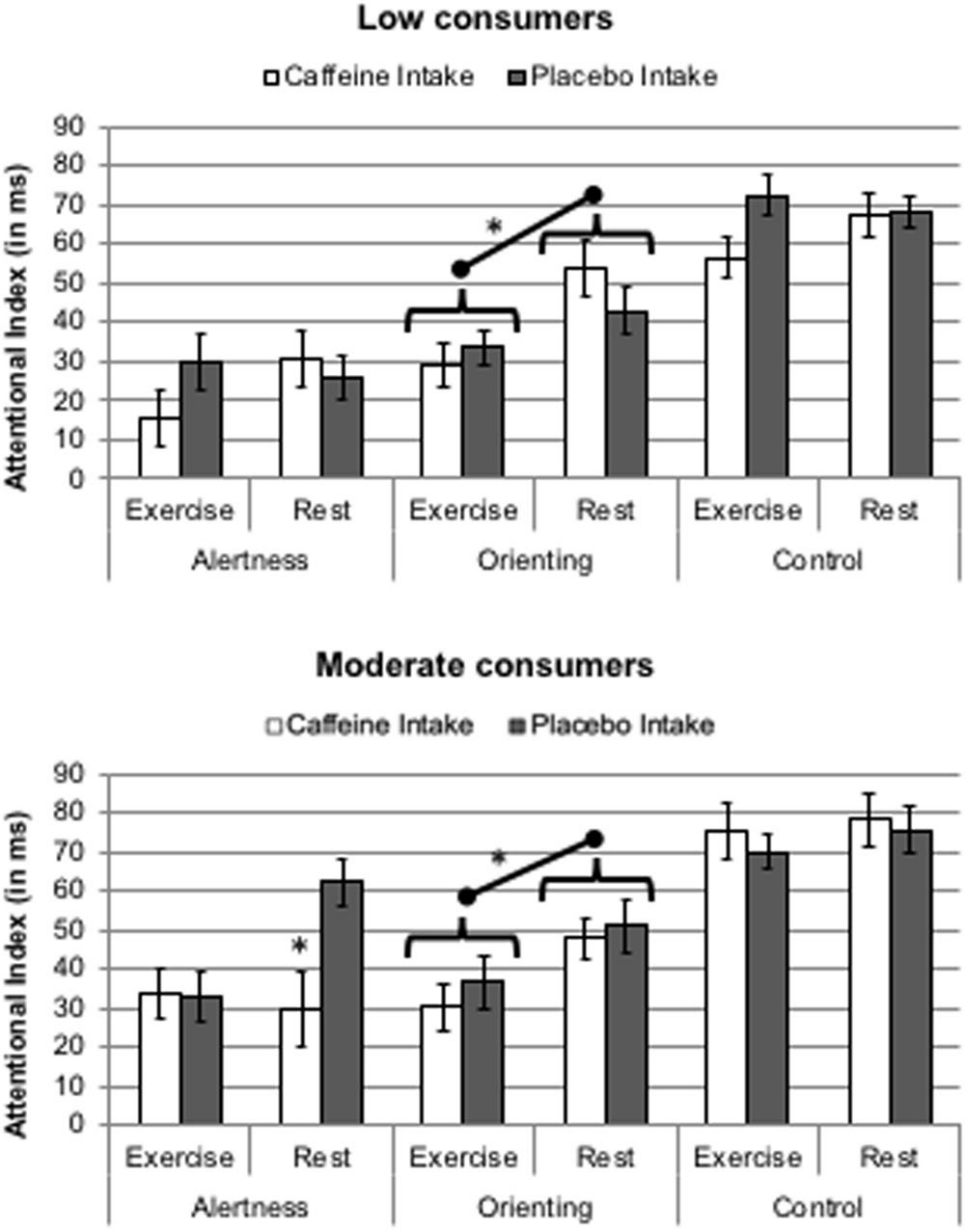
Attentional index of the functioning of each attentional network, as a function of Activity, Caffeine intake and Consum Habit (top panel: Low caffeine consumers; bottom panel: Moderate caffeine consumers). Alertness index = RT No Tone – RT Tone trials (exclusively on No cue trials). Orienting index = RT Uncued – RT Cued trials. Control index (interference) = RT Incongruent – RT Congruent trials. Note: *p < 0.05.

A 2 (Consumption habit) × 2 (Intake) × 2 (Activity) × 2 (Alertness; No Tone vs. Tone) mixed MANOVA was performed on Mean RT. Following previous studies^12^, only data from the no cue condition was considered for this analysis. A main effect of Alertness was observed, *F*(1, 22) = 109.75, *p* < 0.001, η^2^ = 0.83, with 31 ms faster RT for the Tone than the No Tone condition. More importantly, this alertness effect was modulated by both Intake and Activity, *F*(1, 22) = 4.91, *p* = 0.037, η^2^ = 0.18, and *F*(1, 22) = 4.99, *p* = 0.036, η^2^ = 0.18, respectively. The four-way interaction was also significant, *F*(1, 22) = 5.13, *p* = 0.034, η^2^ = 0.19, showing a marginal reduction of alertness effect with caffeine intake (compared with no intake), in low consumers at the exercise condition (see top panel of Fig. 4), *F*(1, 11) = 4.47, *p* = 0.058, η^2^ = 0.29, but a significant reduction in moderate consumers at the rest condition (see bottom panel of Fig. 4), *F*(1, 11) = 5.15, *p* = 0.044, η^2^ = 0.32.

The corresponding analysis of the error percentages only revealed as significant the Intake × Alertness interaction, *F*(1, 22) = 5.74, *p* = 0.026, η^2^ = 0.21. Whereas after caffeine intake slightly more errors (0.61%) were made in the presence of the tone than when it was absent, less errors (0.30%) were made when the tone was present in the placebo intake condition.

#### Orienting

A 2 (Consumption habit) × 2 (Intake) × 2 (Activity) × 2 (Orienting; uncued, no cue, cued) mixed MANOVA was performed on Mean RT. The expected main effect of Orienting was highly significant, *F*(2, 44) = 153.27, *p* < 0.001, η^2^ = 0.87. More interestingly, Orienting was modulated by Activity, *F*(2, 44) = 13.48, *p* < 0.001, η^2^ = 0.38, leading to reduced orienting effects in the exercise condition in both groups, as shown in Fig. 4 (top and bottom panels). Both the costs (uncued minus no cue), *F*(1, 22) = 6.46, *p* < 0.019, η^2^ = 0.23, and the benefits (no cue minus cued), *F*(1, 22) = 10.78, *p* = 0.003, η^2^ = 0.33, were reduced in the exercise (30 and 19, respectively) compared to the rest (22 and 11 ms, respectively) condition.

The corresponding analysis or error percentages also showed a significant mean effect of orienting, *F*(2, 44) = 12.01, *p* < 0.001, η^2^ = 0.35. Additionally, a significant Intake × Orienting interaction was found observing larger orienting effect in the caffeine intake (1.98%) than in the placebo intake (0.80%) condition, *F*(2, 44) = 3.39, *p* = 0.043, η^2^ = 0.13.

#### Control

A 2 (Consumption habit) × 2 (Intake) × 2 (Activity) × 2 (Congruency; Congruent vs. Incongruent) mixed MANOVA was performed on Mean RT. The main effect of congruency was highly significant, *F*(1, 22) = 672.79, *p* < 0.001, η^2^ = 0.97, with 70 ms faster RT for the Congruent than the Incongruent condition. This effect was not modulated by any variable.

The analogous analysis of error percentages revealed a significant main effect of congruency, *F*(1, 22) = 31.22, *p* < 0.001, η^2^ = 0.59. Interestingly, this effect was modulated by Activity, *F*(1, 22) = 8.31, *p* = 0.009, η^2^ = 0.27, with larger interference being observed in the exercise (3.10%) than in the rest (2.16%) condition.

Figure 4 depicts the attentional index of the functioning of each attentional network, as a function of Activity, Caffeine intake and consumer habits.

## Discussion

The present study is the first to measure the effect of caffeine on attention considering its multifunctional nature (alerting, orienting, and executive control) under two different physical effort conditions and using two groups of participants selected according to their caffeine consumption habit. Attentional functioning was assessed under caffeine and placebo intake conditions at rest and during moderate aerobic steady-state exercise.

Previous studies attempting to explore the interactions between exercise and caffeine and their effects on cognition have shown controversial results due to methodological issues: different caffeine dosage and participant’s consumption habit and withdrawal^40^, or moment of cognitive evaluation (before, during or after completion of physical effort^34,59^). Importantly, and considering the recommendations by previous authors, here we tried to control the modulation of different variables that could affect the caffeine effect (individual caffeine dosage and participant’s consumption habits) during exercising.

Our results replicated the general facilitating effect of exercise on speed of response, previously described by similar studies^13,21^, probably related to a shift in the speed-accuracy trade-off toward response speed^44^. More importantly for the aims of the present study, our results have shown rather specific effects of caffeine intake on both reaction speed and the efficiency of the alerting network. Caffeine intake reduced the effect of alerting signals on response speed in low caffeine consumers when they were exercising. Contrarily, for more habitual caffeine consumers caffeine intake only reduced alertness at rest.

Regarding exercise modulations of RT, our results indicated that the applied exercise workload induced a significant enhancement of physiological state (HR and lactate accumulation) in both low and moderate caffeine consumers and, more importantly, that exercise shortened response speed in all experimental conditions, while reduced only slightly the response accuracy. In line with previous accounts, our results replicated the finding about the benefit on response speed during aerobic exercise (below the LT)^5,13,21,60,61^. These results could be partially explained by the arousal theory and increases of adrenaline-noradrenaline secretion induced by exercise^45^. McMorris^44^ has recently described a more comprehensive framework to explain how acute exercise affects cognition from a neurochemical perspective. The author reviewed the litarature showing that intrinsic exercise variables (as duration or intensity) modulate the secretion of different neurochemical factors (catecholamines, cortisol and BDNF). In our study, the slight reduction of response accuracy in the flankers task is in agreement with previous studies^10,44,62^, pointing to the stress level induced by moderate aerobic exercise, which mildly modulates in the sympathetic-adrenal and vagal/nucleus tractus solitarii (NTS) pathway to impair accuracy compared to higher intensities.

Concerning caffeine intake, and similarly to findings included in a recent meta-analysis^63^, our results did not show any main effect of caffeine on HR, lactate accumulation and RT during exercise in any of the controlled experimental conditions. These results are partially incongruent with the resounding evidence of significantly faster RT under caffeine intake conditions due to increase of adrenaline-noradrenaline secretion^64^ using a broad variety of methodological approaches. However, most of these studies underscored the importance of exploring these effects in a range of participant consumption profiles and under different exercising conditions. Importantly, and according to our expectations, the effect of caffeine intake on performance was moderated by both variables (consum habit and exercise), observing that caffeine shortened RT only in moderate caffeine consumers at rest. Yet, importantly, moderate consumers were significantly slower under the placebo-rest condition than low consumers. Our results could be explained by the onset of a withdrawal effect associated with caffeine deprivation in participants more habituated to caffeine consumption (moderate consumers) in conditions where tonic arousal was lower (i.e., the placebo and rest condition), but they equate to non consumers when external arousing stimuli appear (i.e., exercising, caffeine intake or alerting signals). These finding are in agreement with the poorer performance observed under caffeine deprivation of habitual coffee consumers^65,66^. Increases of adrenaline-noradrenaline secretion induced by exercise^45,67^ and caffeine^64^ could justify the greater effect of caffeine on RT under the rest condition, when baseline arousal level is lower.

According to caffeine and exercise modulations of attentional networks, our results replicated the typical pattern of results about the general functioning of the ANTI task in this population (main effects and interactions between attentional networks) on mean RTs and error both at rest^12^ and during exercise conditions^13^. More importantly for the purpose of the current study, our results revealed that the effect of caffeine on phasic alertness was moderated by the consum habit and exercise condition simultaneously. Caffeine intake reduced the effect of alerting signals on RT in low consumers when they were exercising, but contrarily, caffeine induced a lower alertness effect in moderate consumers at the rest condition. In line with previous accounts using a similar attentional tasks, we observed a reduction of alerting acoustic signals effect under exercising^13,25^. This interaction could be expected by taking into account that caffeine interacts with the dopaminergic systems^64^, and that the alertness attentional network activates dense dopaminergic innervated areas^68^. However, and contrarily to Brunyé *et al*. using the ANT, who observed a lower alerting size effect under placebo compared to both the 200 mg and 400 mg caffeine intake conditions in low consumers^37^, our data showed a larger alerting effect under placebo than under caffeine intake conditions. Interestingly, the differences between our results and those obtained by Brunyé and colleagues^36,37^ could be related to the different measure of the alerting function as assessed with the Attentional Network Test (ANT) vs. the ANT-I used here. The ANT uses a less arousing visual cue to measure alertness, whereas the ANT-I uses an auditory cue, which seems to be more effective in producing alertness^69^. Thus, a more arousing alerting cue in our study produced larger alertness in the reduced tonic activation condition (placebo intake), whereas a less arousing cue lead to larger alertness only when added by cafeine intake arousal. This idea is supported by previous findings^36^ suggesting that, given that caffeine upregulates dopaminergic availability, more habituated caffeine consumers may need higher doses to reach a similar “baseline” state of vigilance than less habitual consumers. Taken altogether, our results seem to suggest that higher phasic alertness (due to slower responses in no tone conditions) is usually observed under conditions of reduced tonic vigilance, as in rest and placebo conditions in the present study or morning-type individuals when tested in the evening^46^. According to the observed interaction between all “arousing” variables (alerting stimulus, activity and caffeine intake), our results are coherent with the hypothesis whereby the locus coeruleus–norepinephrine system is activated in a similar manner by these variables^70^.

Concerning the orienting network, results revealed that, compared to the rest condition, only exercise modulated this function inducing a decrease in spatial attentional orienting, both in attentional benefits and attentional costs. Our results are partially consistent with others showing a reduction of the spatial orienting effect^21^, and revealing a deployment of exogenous spatial attention after a bout of intense aerobic exercise^22^. Present results may indicate that exercise-induced physiological changes activate the attentional system, making it more reactive to relevant peripheral stimuli, and therefore being less affected by irrelevant cues. However, our finding is somewhat in contrast with other study using the ANT-I task during an aerobic exercise in a group of highly skilled cyclists and did not observe an interaction between exercising and exogenous visual spatial attention^13^. These controversial results could be explained by previous findings showing that exercising affect spatial visual orienting differently depending on the participant’s physical fitness level^22^ and motor expertise^71^. Further research investigating the exercise-attentional networks functioning interactions should consider the role of both moderators (physical fitness and/ or motor skills).

Regarding the effect of caffeine on the orienting function, we replicated previous results observed in high consumers by Brunyé and colleagues^37^ showing that caffeine did not modulate the orienting network function when response speed was considered, although they found a marginal effect of caffeine on orienting in low consumers when using larger doses than the ones used in the present study. Overall, these results support the idea that caffeine-induced changes in orienting may depend on the caffeine doses used and individuals’ consumption habit. The absence of a significant interaction between caffeine and visual orienting has been explained by arguing that the brain areas involved in attentional orienting (superior parietal lobe brain areas)^72^ have sparse dopaminergic innervation, while dopamine is the neurotransmitter affected by caffeine.

In regard to the executive control network our results are in line with previous findings^9,13^ showing no evidence of any modulation of executive control performance by exercise. However, regarding the effect of caffeine on executive functioning, our results challenge those previously described by Brunyé and colleagues demonstrating that the group of low-caffeine consumers exhibited dose-dependent increases in the executive control function (at doses from 0 to 400 mg)^36,37^. Yet, the daily caffeine intake of participants almost doubled the intake of the low consumers in our study.

## Conclusions

Our results support the assumption that exercise speeds up response ability by modulating exogenous stimulus-driven attentional functions (i.e., alerting and exogenous spatial orienting). These findings could be explained in terms of exercise-induced increases in physiological arousal and consequently a better efficiency of peripheral motor processes during exercise conditions. Nevertheless, this hypothesis should be verified by further research, for instance, by exploring the relationship between electromyographic response and attentional networks functioning during exercise.

Importantly, our results show that caffeine and exercise have a similar effect on performance, speeding up motor responses, but this effect is moderated by the caffeine consume habit. In this regard, habitual caffeine intake induces a reduction of tonic arousal, which turns comparable to the level in non-consumers when other arousing stimulus appear (exercise, caffeine intake or alerting signals).

Given the interaction between these two variables (activity and caffeine), we can assume that they both increase general activation in a similar way. However, the absence of interactions between Intake and Activity with regard to orienting and executive control suggests that exercise and caffeine are different modulators of attention. Only in the case of the modulation over the general activation indexed by alertness these two variables seems to interact. It seems that each attentional network, depending on its behavioral significance, is modulated differently by internal and external “arousing” conditions.

Overall, the results of this study suggest that caffeine intake seems to be necessary in more habituated caffeine consumers to reach an optimal level of general arousal and respond in low “arousing” situations (i.e., rest conditions). Caffeine deprivation periods in more habitual consumers may impair RT performance due to lower general activation at baseline related to dopamine availability conditions.

The systematic control and administration of acute and habitual personalized caffeine doses is relevant in order to optimize the balance between physiological and cognitive cost and benefits. These recommendations could be extended to different contexts where caffeine consumption is habitual to cope with the high demands of vigilance and accurate decision making during prolonged time, as in professional drivers, pilots or air traffic controllers.

## Data Availability

The datasets generated during and/or analysed during the current study are available in the OSFHOME repository, DOI 10.17605/OSF.IO/VZ6NH, https://osf.io/vz6nh/?view_only=a7ee034bb2cf47ba8f4b3f0bf6a139e9.
